# On Cyclic-Fatigue Crack Growth in Carbon-Fibre-Reinforced Epoxy–Polymer Composites

**DOI:** 10.3390/polym16030435

**Published:** 2024-02-04

**Authors:** Silvain Michel, Neal Murphy, Anthony J. Kinloch, Rhys Jones

**Affiliations:** 1 Empa, Swiss Federal Laboratories for Material Science and Technology, Laboratory for Mechanical Systems Engineering, CH-8600 Dübendorf, Switzerland; silvain.michel@empa.ch; 2 School of Mechanical & Materials Engineering, University College Dublin, D04 C1P1 Dublin, Ireland; neal.murphy@ucd.ie; 3 Department of Mechanical Engineering, Imperial College London, Exhibition Road, London SW7 2AZ, UK; a.kinloch@imperial.ac.uk; 4 Department of Mechanical and Aerospace Engineering, Monash University, Clayton, VIC 3800, Australia

**Keywords:** crack propagation, delamination growth, fatigue, fracture mechanics, Hartman–Schijve, polymer composites, simple scaling

## Abstract

The growth of cracks between plies, i.e., delamination, in continuous fibre polymer matrix composites under cyclic-fatigue loading in operational aircraft structures has always been a very important factor, which has the potential to significantly decrease the service life of such structures. Whilst current designs are based on a ‘no growth’ design philosophy, delamination growth can nevertheless arise in operational aircraft and compromise structural integrity. To this end, the present paper outlines experimental and data reduction procedures for continuous fibre polymer matrix composites, based on a linear elastic fracture mechanics approach, which are capable of (a) determining and computing the fatigue crack growth (FCG) rate, *da*/*dN*, curve; (b) providing two different methods for determining the mandated worst-case FCG rate curve; and (c) calculating the fatigue threshold limit, below which no significant FCG occurs. Two data reduction procedures are proposed, which are based upon the Hartman-Schijve approach and a novel simple-scaling approach. These two different methodologies provide similar worst-case curves, and both provide an upper bound for all the experimental data. The calculated FCG threshold values as determined from both methodologies are also in very good agreement.

## 1. Introduction

The growth of interlaminar cracks, i.e., delamination, between the plies in polymer matrix continuous fibre composites under cyclic-fatigue loading in operational aircraft structures, and in other industries such as the building sector, has always been a very important factor and is known to have the potential to significantly decrease the service life of a composite airframe. Indeed, as explained in the US DoD composite materials design guidelines, CMH-17-3G [1], current aircraft designs are based on a ‘no growth’ design philosophy. However, delamination damage can nevertheless arise in operational aircraft and compromise structural integrity. A number of examples of this are provided in [2,3]. When assessing the service life of an airframe, the US Joint Services Structural Guidelines JSSG-2006 [4] specifically require that the structure is subjected to a full-scale fatigue test that is equal to, or greater than, twice the design life of the aircraft. This means that, for any small initial delamination that is inherent in the structure, or for delamination damage found in service, the crack driving force should ideally be beneath the value of the fatigue threshold limit, below which no significant FCG occurs. If not, then, in accordance with MIL-STD-1530D [5], delamination growth should be slow, and any delamination present in the structure must not grow to the point where it causes failure in under two lifetimes. These requirements highlight the importance of using fatigue threshold values in the design of composite airframes. These statements also need to be taken in conjunction with (a) the statement in NASA Fracture Design Handbook NASA-HDBK-5010 [6] that mandates the use of a ‘worst-case upper bound’ fatigue crack, i.e., delamination, growth curve, and (b) the statement in MIL-STD-1530D [5] that mandates the use of a linear elastic fracture mechanics (LEFM) approach [7,8,9]. Finally, it is noteworthy that, as explained in MIL-STD-1530D [5], the role of full-scale testing is merely to validate or correct analysis methods and to demonstrate that the stated requirements are achieved. As such, a means for determining the worst-case delamination growth curve and the corresponding worst-case fatigue threshold is an essential requirement for both design and through-life sustainment.

Furthermore, before any LEFM analysis of an in-service structure can be undertaken, it is essential that the fatigue crack growth (FCG) curve for delamination propagation in the continuous carbon-fibre polymer matrix composite is accurately and reproducibly assessed from laboratory test specimens, and that the data generated can be directly related to real-life structures, i.e., the principle of ‘similitude’ is acting [10,11]. However, in this context, it should be noted that previous work, e.g., refs. [12,13], has shown that a series of FCG curves for a given continuous carbon fibre epoxy polymer composite are often determined in laboratory tests, as opposed to just one such unique FCG curve. These different FCG curves reflect different extents of fibre bridging, which develops behind the advancing crack tip, present in the test specimen. The development of fibre bridging in a fatigue test will limit the degree of delamination that occurs and give the false impression of relatively superior fatigue behaviour. Such a series of FCG curves can be generated by simply varying the pre-crack length, *a_p_-a_o_*, in the test specimen prior to the start of the laboratory cyclic-fatigue test. On the other hand, delamination under cyclic loading in an aircraft structure typically involves limited or no fibre bridging [14]. Hence, there is little retardation of the FCG rate in real structures. As a result, to meet the requirement that laboratory test data are able to be translated to an operational airframe, and that any assessment of delamination growth be based on the worst-case FCG curves and the worst-case fatigue threshold, it is necessary that these various FCG curves, which are a function of the pre-crack length, *a_p_-a_o_*, as measured in the laboratory, are reproducible and that the effects of fibre bridging can be assessed and eliminated. Only then will the principle of similitude be acting.

Consequently, in the present article, we first present experimental data obtained at the Swiss Federal Laboratories for Materials Science and Technology (Empa, Dübendorf) and at University College Dublin (UCD, Ireland) as part of a European Structural Integrity Society (ESIS), Technical Committee (TC) 4 (on Polymer, Composites and Adhesives) round-robin study into the reproducibility of delamination growth under cyclic-fatigue loads. Secondly, the measured FCG rate, *da*/*dN*, data, as a function of the pre-crack length, *a_p_-a_o_*, prior to the start of the laboratory cyclic-fatigue test are discussed and compared. Thirdly, the major aims of the present work are to develop data reduction procedures that are capable of (a) accurately determining and computing the FCG rate, *da*/*dN*, curves; (b) providing methods for determining the worst-case, upper-bound FCG rate curve; and (c) providing methods for determining the worst-case fatigue threshold value. As noted above, such FCG rate curves and fatigue thresholds are essential for (a) sound material development, characterisation, and comparison studies and (b) accurate design and lifespan studies, where their use is indeed mandated. To achieve these aims, two different methodologies are proposed. One is based upon the Hartman-Schijve equation [13,14,15,16,17,18], while the other is based on a novel simple-scaling equation. These approaches are employed to compute the experimentally measured FCG curves as a function of the pre-crack length, and to determine the worst-case, upper-bound FCG curves and the corresponding fatigue threshold limit values for the FCG curves. Results from the two different methodologies are compared with one another and to the experimental results.

## 2. Materials and Methods

### 2.1. Material and Test Specimen

For the carbon-fibre reinforced-plastic (CFRP) material, a continuous fibre epoxy matrix polymer composite was employed, which was fabricated using a prepreg of unidirectional, continuous carbon fibres in a thermosetting epoxy polymer matrix (‘M30SC/DT120′ supplied by Delta-Tech S.p.A., Altopascio, Italy). (A unidirectional, continuous carbon-fibre composite was chosen since this is the orientation of the fibres given in the ISO Standard test method [19] and this fibre orientation typically results in the most severe degree of fibre-bridging occurring). Panels of CFRP were produced of this composite and were manufactured using a hand lay-up by the Delft University of Technology, the Netherlands, with a lay-up sequence of [(0)_16_//(0)_16_] to give a nominal thickness of 5 mm. In all cases, a 12.7 µm thick film of poly(tetrafluoroethylene) (‘Teflon’, Du Pont, Wilmington, DE, USA) was inserted into the mid-plane of the CFRP panel at one end, normal to the 0° fibre direction, during the hand lay-up process to act as an initial delamination, or ‘starter crack’, of a nominal length, *a_o_*, of 50 mm. The laid-up panels were placed in an autoclave and cured for 90 min at a temperature of 120 °C under a pressure of 6 bar. The glass transition temperature of the resulting thermosetting epoxy polymer matrix was 120 °C.

### 2.2. Test Method

**Figure 1:**
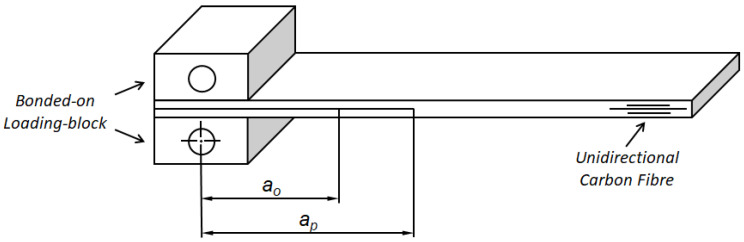
Sketch of the double cantilever beam (DCB) continuous carbon-fibre epoxy polymer matrix composite test specimen. Showing the initial, starter-crack, delamination of length, *a_o_*, in the DCB specimen, which was introduced to a pre-crack length of *a_p_* before measurements were taken for the cyclic-fatigue test.

For the cyclic-fatigue tests, the Mode I (opening tensile mode) double cantilever beam (DCB) test specimen was used [19], as shown in Figure 1. In order to produce these specimens, the cured CFRP panel was cut into strips nominally 200 mm × 20 mm, with the long direction aligned with the 0° fibre direction. End blocks were first adhesively bonded, with a nominal adhesive thickness of 0.5 mm, onto the end of the DCB specimen, where the starter film had been inserted, in order to apply cyclic loads to the specimen.

The cyclic-fatigue tests were undertaken according to the test protocol outlined in [15,20]. Prior to measurements from the cyclic-fatigue test being taken, an initial, starter-crack delamination of length *a_o_* in the DCB specimen was grown to a pre-crack length of *a_p_* to form a natural crack front under quasi-static loading of the specimen (see Figure 1). It should be noted that if a pre-crack extension length of *a_p_-a_o_* is not used in the fracture mechanics test, then optimistically high values of the toughness and fatigue resistance will be measured. This is because the starter crack film of length *a_o_* represents a relatively blunt crack tip compared to that of a pre-crack of length *a_p_*, which is naturally-grown ahead of the starter film prior to the commencement of the test.

Next, for any given DCB specimen, the test method that was employed involved growing the crack under cyclic-fatigue loading, at a relatively low frequency of 5 Hz to avoid heating effects, for a relatively short distance from the initial value of the pre-crack extension length, *a_p_-a_o_*, whilst taking readings of the number of cycles, *N*, crack length, *a*, load, *P*, and displacement, *δ*. The fatigue test was then halted and repeated, but now with respect to the new, longer crack that was present in the DCB specimen, i.e., the crack length *a_p_-a_o_* that was now present was taken to be the relevant value for this repeated fatigue test. Thus, a key variable when testing a given DCB test is the value of the pre-crack extension length, *a_p_-a_o_*, prior to measurements being taken for any cyclic-fatigue test. For these fatigue tests, the value of the R-ratio used was 0.1; here, the R-ratio was defined as $R=P_{min}/P_{max}$, where $P_{min}$ and $P_{max}$ are the minimum and maximum loads, respectively, that were applied during the fatigue test. Displacement control was used for these fatigue tests. The measured data were employed to calculate, for a given DCB test at a given value of the crack extension length, *a_p_-a_o_*, the FCG rate, *da*/*dN*, using the ‘incremental polynomial method’ according to the ASTM Standard [15,20,21]. Also, the values of the maximum and minimum energy release rate, $G_{max}\;$and $G_{min}$, applied in a fatigue cycle were deduced from the maximum and minimum applied measured loads,$\;P_{max}$ and $P_{min},$ respectively, using the ‘modified compliance method’ as described in detail in the ISO Standard [19].

Three DCB fatigue tests were undertaken at the Empa laboratory, designated as tests 1.1.4 to 1.1.6, using various values of the pre-crack extension length of *a_p_-a_o_*. Four DCB fatigue tests were undertaken at the UCD laboratory, designated as tests 2.1.3 to 2.1.6, again using various values of the pre-crack extension length.

## 3. Theoretical Aspects

### 3.1. Overview

The first important decision is the parameter against which to plot the measured FCG rate, *da*/*dN* (see Section 3.2 below). Next, three major aims of the present work were to develop data reduction procedures capable of (a) accurately determining and computing the FCG rate, *da*/*dN*, curves, (b) providing methods for determining the worst-case, upper-bound FCG rate curves, and (c) providing methods for determining the worst-case fatigue threshold value. Such FCG rate curves are essential for (a) sound material development, characterisation, and comparison studies, and (b) accurate design and lifing studies where their use is indeed mandated. Two different methodologies are proposed in the present paper based upon the Hartman-Schijve equation [13,14,15,16,17,18] and a novel simple-scaling equation. These are discussed in Section 3.3 and Section 3.4, respectively.

### 3.2. The Appropriate Crack-Tip Parameters

When Paris and co-workers originally formulated the equations for FCG in metals [9,22,23], they argued that, since Irwin [8,24] had shown that the stress-intensity factor, K, uniquely characterises the near tip stress field, then the rate of FCG, i.e., *da*/*dN*, should be a function of ∆K and $K_{max}$. Here, the range of the stress-intensity factor, ∆K, is given by $∆K=K_{max}-K_{min}$, where these two terms represent the maximum and minimum of the applied stress-intensity factor in a fatigue cycle, respectively. Subsequently, Sih, Paris, and Irwin [25] extended the Irwin solution for the crack-tip stress field to rectilinearly orthotropic composites. Their solution revealed that the near-tip stress field for rectilinearly orthotropic composites was uniquely described by $\sqrt{G}$. As a result, [26] suggested that the logical extension of the Paris FCG law for metals to delamination growth in continuous fibre polymer composites is to express *da*/*dN* as a function of $\sqrt{G_{max}}$ or $∆\sqrt{G}$, and not $G_{max}$ nor ∆G. Thus, the logical extension of the Paris growth law to such composites is to express *da*/*dN* as a function of $\sqrt{G_{max}}$ or $∆\sqrt{G}$. Further, as shown in [27], expressing *da*/*dN* as a function of ∆*G* often leads to the anomalous conclusion that, for a given ∆*G*, increasing the mean stress level reduces the FCG rate. This anomaly is removed if *da*/*dN* is expressed as a function of $∆\sqrt{G}$ (see [27]). Consequently, in the present paper, the FCG rate, *da*/*dN*, is plotted as a function of$\;∆\sqrt{G}$, where the term $∆\sqrt{G}\;$is given by:

(1) $$\;∆\sqrt{G}=\sqrt{G_{max}}-\sqrt{G_{min}}$$

where $G_{max}$ and $G_{min}\;$are the maximum and minimum energy release rates in a fatigue cycle, respectively.

### 3.3. The Hartman-Schijve Methodology 

For FCG in metals, the Hartman-Schijve equation [13,14,15,16,17,18], which is a variant [28] of the NASGRO equation, is often employed. The Hartman-Schijve methodology (a) allows for any effects in the FCG curves due to the values of $K_{max}$ and $K_{min}/K_{max}$, (b) provides a similitude parameter so that the FCG curves from a test specimen are directly comparable to the FCG seen in a real component, (c) enables the worst-case, upper-bound FCG curve to be determined, (d) enables the worst-case fatigue threshold value also to be deduced, and (e) takes into account the statistical scatter observed in the material and in the experimental test data on the values for the worst-case, upper-bound FCG curve and threshold value. 

In terms of$\;∆\sqrt{G}$, the Hartman-Schijve equation may be expressed as in [11,13,14,15,16,17,18]:

(2) $$\frac{da}{dN}=D{\left[\frac{∆\sqrt{G}-∆\sqrt{G_{thr}}}{\surd \left\{1-\sqrt{G_{max}}/\surd A\right\}}\right]}^{n}$$

where *D* and *n* are constants and *A* is the cyclic fracture toughness. The term $∆\sqrt{G_{thr}}$ is defined by

(3) $$∆\sqrt{G_{thr}}=\sqrt{G_{thr.max}}-\sqrt{G_{thr.min}}$$

and the subscript ‘*thr*’ in Equations (2) and (3) refers to the values at the threshold limit, below which no significant FCG occurs. (The relationship between $∆\sqrt{G_{thr}}$ and the ASTM defined fatigue threshold, $∆\sqrt{G_{th}}$, which is arbitrarily chosen as the value of $∆\sqrt{G}$ at a crack growth rate of 10^−10^ m/cycle, is presented in the Appendix A). As a matter of interest, Equation (2) leads to the crack driving force, ∆*κ*, being defined [29] as

(4) $$∆\kappa =\left[\frac{∆\sqrt{G}-∆\sqrt{G_{thr}}}{\surd \left\{1-\sqrt{G_{max}}/\surd A\right\}}\right]$$

The term ∆*κ* is of some importance since it has been shown [11,27] to represent a valid ‘similitude’ parameter. This topic is also discussed in the Appendix A.

The first step in the proposed Hartman-Schijve methodology is to replot the measured values of *da*/*dN* versus $∆\sqrt{G}$ in the form of Equation (2) to obtain a single, linear, unique, ‘master’ plot of logarithmic *da*/*dN* versus logarithmic $\left[\frac{∆\sqrt{G}\;-\;∆\sqrt{G_{thr}}}{\surd \left\{1\;-\;\sqrt{G_{max}}/\surd A\right\}}\right]$. Previous work [11,13,14] has shown that this may be readily achieved by selecting values of $∆\sqrt{G_{thr}}$ and *A* to ensure that Equation (2) fits all the experimental data over the entire range of measured FCG rates. Indeed, it has been found that a single, linear, unique master representation may be observed for CFRP composites by allowing for relatively small changes in the values of $∆\sqrt{G_{thr}}.\;$Such a linear master representation also yields the values of *D* and *n*. Next, the values of *da*/*dN* versus $∆\sqrt{G}$ may be computed according to the Hartman-Schijve equation (see Equation (2)) and compared with the experimentally measured values. Finally, from the mean and standard deviation values of $∆\sqrt{G_{thr}}$, a worst-case, upper-bound FCG rate curve may now readily be calculated by using Equation (2) and taking the mean value of $∆\sqrt{G_{thr}}$ minus three standard deviations. As explained in detail in [30,31], the value of three times the standard deviation is mandated for this calculation since the value of three times the standard deviation represents the allowable scatter for designing and lifing a single CFRP member where the loading is such that its failure would result in a loss of structural integrity of the component. Therefore, in the present paper, we have chosen, as mandated in NASA-HDBK-5010 [6], to use the mean value of $∆\sqrt{G_{thr}}$ minus three standard deviations to calculate the worst-case, upper-bound FCG curve.

### 3.4. The Simple-Scaling Methodology 

Several authors [27,32,33,34,35,36,37,38,39] have suggested that a simple scaling, involving a normalisation of the energy release rate term against which the FCG rate data are plotted, may reduce the differences seen in the FCG rate curves, regardless of whether such differences arise from scatter in the experimental data and/or from the development of fibre bridging as the fatigue crack propagates, i.e., as the value of *a_p_-a_o_* is increased. 

Poursartip [32] was the first to suggest that delamination growth rate in composites could be normalised, when he considered the maximum value of the applied energy release rate, $G_{max}$, in the fatigue cycle, by using the expression $G_{max}/G_{R}(a)$, where $G_{R}(a)$ is the quasi-static fracture energy as a function of the length of the propagating crack (since, due to the fibre bridging that develops in continuous carbon-fibre epoxy polymer composites, the measured toughness in a quasi-static test is also typically a function of the crack length as the delamination propagates). This scaling approach to represent the FCG behaviour, which is entirely empirical, is now moderately widely used, e.g., [27,32,33,34,35,36,37,38,39], and has the advantage that it may significantly reduce the data scatter. 

However, it seemed to the present authors from their previous work [27] that such a normalising term should more logically include a threshold term, i.e., taken at a relatively very low value, with the aim of using a simple-scaling methodology to not only reduce the scatter but also to determine the FCG curve and identify a threshold limit at which there is no significant FCG. If this new simple-scaling approach is successful in achieving these goals, then it provides a more direct and rapid methodology than the Hartman-Schijve methodology described above. To this end, a primary objective of the present study was to develop a simple-scaling approach that was unrelated to the Hartman-Schijve methodology and thereby allowed an independent assessment of both approaches.

The novel simple-scaling equation that has been used in the present study involves two steps. In the first step, for each value of *a_p_-a_o_*, the FCG rate, *da*/*dN*, is plotted against a normalised *x*-axis of $∆\sqrt{G}/∆\sqrt{G_{da/dN}}$. Here, $∆\sqrt{G}$ is the experimentally measured value (see Equation (1)) and the normalising term is $∆\sqrt{G_{da/dN}}$, which is the value of $∆\sqrt{G\;}$ taken from the measured FCG curves for a given, relatively low value of *da*/*dN*. In the present study, a value of *da*/*dN* that is equal to 10^−8^ m/cycle was chosen. (It should be noted that choosing a value of 10^−8^ m/cycle was arbitrary and any relatively low value of *da*/*dN* could equally well have been taken with no significant effect on the resulting normalised FCG curves occurring. This particular value of *da*/*dN* was chosen since it represented the lowest value of *da*/*dN* that was common to all of the data). As shown in [27], this approach has the potential to collapse the various *da*/*dN* versus $∆\sqrt{G}$ curves, which are dependent on the value of *a_p_-a_o_*, onto a single *da*/*dN* versus $∆\sqrt{G}/∆\sqrt{G_{da/dN}}$ curve. That is, in a first approximation, the *da*/*dN* versus the normalised $∆\sqrt{G}$ curves are now independent of the value of *a_p_-a_o_*. This enables the determination of an equation for the associated best fit for the unique *da*/*dN* versus $∆\sqrt{G}/∆\sqrt{G_{da/dN}}$ curve. In the second step, noting that, as discussed in [40], the quasi-static fracture energy for crack initiation, $G_{co}$, represents the highest possible value of $G_{max}$, an estimate of the worst-case crack growth curve is then obtained using the following equation:

(5) $$∆\sqrt{G^{*}}=SCF.\left[\frac{∆\surd G}{\;∆\sqrt{G_{da/dN}}}\right]$$

The term SCF is a scaling factor that is a constant for a given set of tests for a given composite material. It is a function of *R* and $G_{co}$ but is independent of the value of *a_p_-a_o_*. The role of the SCF term is to fix the position of the worst-case FCG curve via this simple-scaling methodology when the *x*-axis is expressed as $∆\sqrt{G^{*}}$, so that this worst-case curve may be compared to that from the Hartman-Schijve methodology and to the experimental results. The value of SCF is calculated using the above best-fit equation, such that at an FCG rate *da*/*dN* of 10^−2^ m/cycle, the value of $\Delta \sqrt{G^{*}}$ is equal to the worst-case, i.e., mean–3*σ*, value of ${\left(1-R\right)G}_{co}$. Precise details on how this is performed for the various UCD and Empa tests examined in the present paper are given below in Section 4.6.

## 4. Results and Discussion

### 4.1. Overview

**Figure 2:**
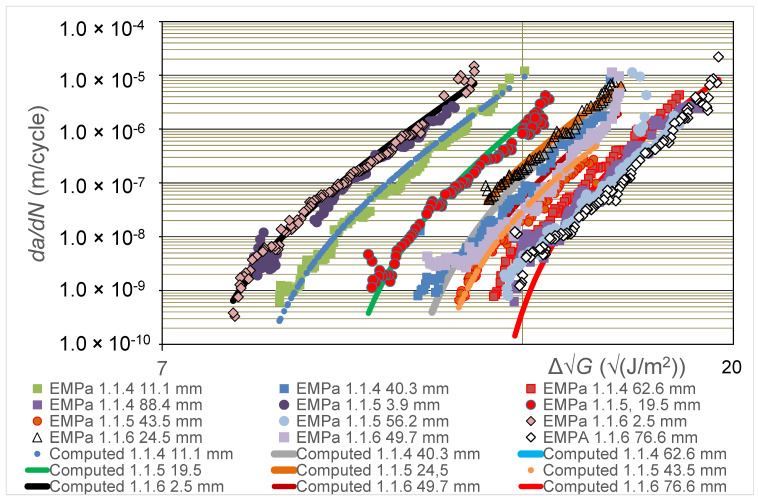
Values of logarithmic *da*/*dN* versus logarithmic $∆\sqrt{G}$ from the tests performed at Empa for the carbon-fibre epoxy polymer composite. Values are given in the legend for the pre-crack extension length, *a_p_-a_o_*, prior to the start of measurements from the DCB fatigue test. (Also shown are the computed relationships from using Equation (2) with the values of $∆\sqrt{G_{thr}}$ and *A* as determined from fitting the experimental results to give the Hartman-Schijve linear, master relationship, as shown later in Figure 5).

**Figure 3:**
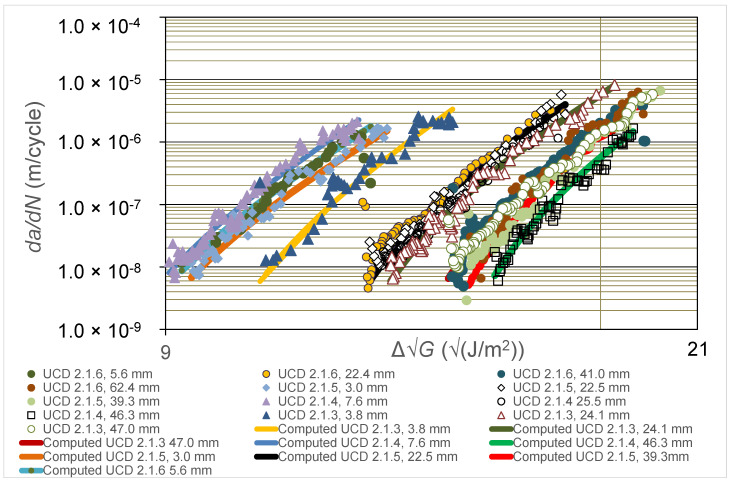
Values of logarithmic *da*/*dN* versus logarithmic $∆\sqrt{G}$ from the tests performed at UCD for the carbon-fibre epoxy polymer composite. Values are given in the legend for the pre-crack extension length, *a_p_-a_o_*, prior to the start of measurements from the DCB fatigue test. (Also shown are the computed relationships from using Equation (2) with the values of $∆\sqrt{G_{thr}}$ and *A* as determined from fitting the experimental results to give the Hartman-Schijve linear, master relationship, as shown later in Figure 5).

**Figure 4:**
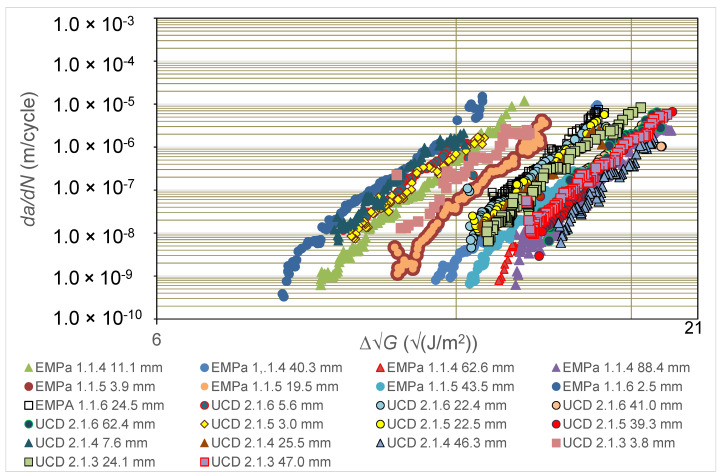
Values of logarithmic *da*/*dN* versus logarithmic $∆\sqrt{G}$ from the tests performed at Empa compared to those performed at UCD for the carbon-fibre epoxy polymer composite. Values are given in the legend for the pre-crack extension length, *a_p_-a_o_*, prior to the start of measurements from the DCB fatigue test.

**Figure 5:**
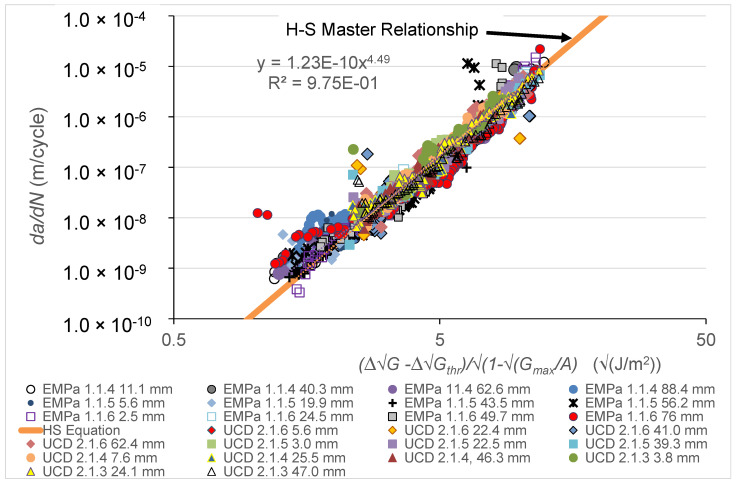
The linear, master relationship obtained for all the Empa and UCD tests for the carbon-fibre epoxy polymer composite as calculated using the Hartman-Schijve (H-S) methodology, i.e., Equation (2).

**Figure 6:**
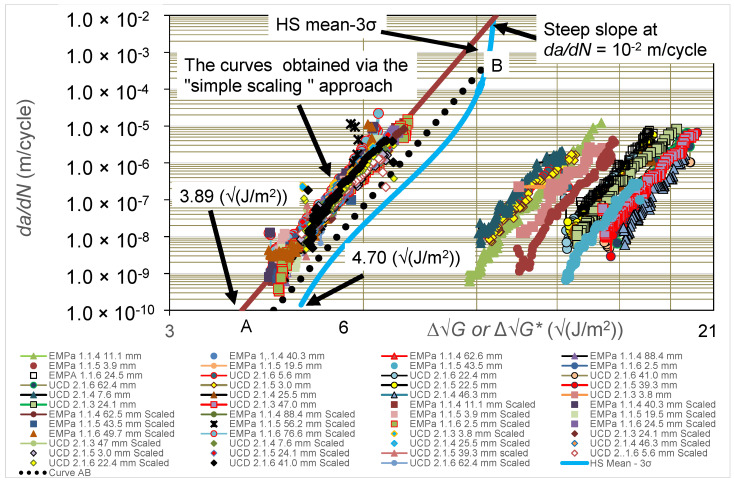
Values of logarithmic *da*/*dN* versus logarithmic $∆\sqrt{G}$ from the tests performed at Empa and UCD for the carbon-fibre epoxy polymer composite. Values are given in the legend for the pre-crack extension length, *a_p_*-*a_o_*, prior to the start of measurements from the DCB fatigue test. The worst-case, upper-bound curve for the FCG rate calculated from the Hartman–Schijve methodology (see Equation (2)) is shown in blue, which uses the ‘mean-3*σ*’ values for $∆\sqrt{G_{thr}}$ and *A* (= $G_{co}$) (see Table 1). The data points from the simple-scaling methodology (see Equation (5)) are also shown with a best-fit line drawn through them. (See text for explanation of ‘Curve AB’, shown as black dots).

In the present section, the experimental results from the two different laboratories taking part in the comparative round-robin study, i.e., Empa and UCD, are first discussed (see Figure 2, Figure 3 and Figure 4). The use of the Hartman-Schijve Equation (2) to replot all these data to obtain a unique, linear, master relationship is then presented (see Figure 5), and the parameters deduced from this approach are used to compute the experimentally measured results that are shown in Figure 2 and Figure 3. Furthermore, the ‘worst-case upper-bound curve’ that may be calculated from the Hartman-Schijve methodology is then deduced and is compared to the experimentally measured results (see Figure 6). The alternative approach of using the novel simple-scaling methodology (see Equation (5)) is then explored and the results are compared in Figure 6 to the worst-case, upper-bound curve for the FCG rate calculated from using the Hartman-Schijve methodology (see Equation (2)) for the CFRP epoxy polymer composite. 

### 4.2. The Experimentally Measured FCG Curves

In Figure 2 values of the logarithmic *da*/*dN* versus the logarithmic $∆\sqrt{G}$ calculated from the tests performed at the Empa laboratory for the CFRP epoxy polymer composites are given as a function of the pre-crack extension length, *a_p_-a_o_*, prior to the start of measurements from the DCB fatigue test. Three replicate DCB tests were undertaken and the testing for each DCB specimen involved growing the crack under cyclic-fatigue loading for a relatively short distance from the initial value of the pre-crack extension length, *a_p_-a_o_*, whilst taking readings of the number of cycles, *N*, crack length, *a*, load, *P*, and displacement, *δ*. The fatigue test was then halted and repeated, but now with respect to the new, longer crack that was present in the DCB specimen, i.e., the longer crack length *a_p_-a_o_* that was now present was taken to be the relevant value for this repeated fatigue test. Similar results were obtained from tests performed at the UCD laboratory for four replicate DCB test specimens and are given in Figure 3, where again the logarithmic *da*/*dN* versus the logarithmic ∆√*G* calculated from the tests for the CFRP epoxy polymer composites are given as a function of the pre-crack extension length, *a_p_-a_o_*, prior to the start of measurements from the DCB fatigue test. 

As may be seen from the results shown in Figure 2 and Figure 3, the FCG curves move steadily to the right as the pre-crack extension length, *a_p_-a_o_*, prior to measurements being taken for the test is increased, until at relatively high values of *a_p_-a_o_*, the FCG rate curves approximately coincide. This implies that there is a retardation of the FCG rate as the value of *a_p_-a_o_* is increased. That is, as the value of *a_p_-a_o_* increases, and for a given value of ∆√*G*, the corresponding value of *da*/*dN* is lower; so, at a given value of ∆√*G,* the fatigue crack grows slower as the value of *a_p_-a_o_* is increased. These observations mainly arise from the extent of fibre bridging in the DCB test specimen being more extensive as the pre-crack extension length, *a_p_-a_o_*, is increased, since this retards the rate of delamination growth at a given value of$\;∆\sqrt{G}$. However, at relatively high values of *a_p_-a_o_*, the FCG rate curves coincide since the extent of fibre bridging has now reached a steady-state saturation level. It is very important to note that such results as in Figure 2 and Figure 3, which agree in general form with those from previous [12,13,14] studies, clearly reveal that there is not one such unique FCG rate curve. Instead, a number of such FCG rate curves may be obtained depending upon the test parameters chosen to run the widely used DCB test. In particular, the chosen value of the pre-crack extension length, *a_p_-a_o_*, at the start of the test, prior to measurements being taken from that given cyclic-fatigue test, is clearly a key test parameter. However, the FCG rate curves do not always move steadily to the right as the pre-crack extension length, *a_p_-a_o_*, prior to measurements being taken for the test is increased, although clearly this is the general trend. Instead, there are a very small number of discrepancies. For example, in Figure 3, it may be seen that the FCG rate curve for a pre-crack extension length, *a_p_*-*a*_o_, of 7.6 mm lies to the left of the curve for a value of *a_p_-a_o_* of 3.0 mm, when it would be expected to lie to the right of the 3.0 mm curve. Such minor discrepancies undoubtedly arise from the inherent scatter in both the composite material and in the experimental testing.

Figure 4 directly compares the experimental results from the Empa and UCD laboratories and several interesting observations arise. Firstly, the results reveal that there is the general trend that the FCG curves move steadily to the right as the pre-crack extension length, *a_p_-a_o_*, prior to measurements being taken for the test is increased, until at relatively high values of *a_p_-a_o_*, the FCG rate curves approximately coincide. As is discussed above, these observations mainly arise from the extent of fibre bridging in the DCB test specimen being more extensive at the start of the test as the pre-crack extension length, *a_p_-a_o_*, is increased, since this retards the rate of delamination growth at a given value of$\;∆\sqrt{G}$. Secondly, the results from replicate tests at a given value of *a_p_-a_o_* for any given laboratory are generally in good agreement, as are such results from the two different laboratories. For example, in Figure 4, compare the results from UCD at values of *a_p_-a_o_* of 22.4 and 22.5 mm and the Empa and UCD tests at *a_p_-a_o_* values of 62.4 and 62.6 mm, respectively. Thirdly, however, as noted above, the FCG rate curves do not always move progressively to the right as the pre-crack extension length, *a_p_-a_o_*, is increased, although clearly this is the general trend. Instead, there are a very small number of discrepancies, which reflects the inherent scatter in both the composite material and in the experimental testing. For example, consider the data points for the UCD tests with an *a_p_-a_o_* value of 3.8 mm, which lie to the right of the UCD tests with an *a_p_-a_o_* value of 3.0 mm, as expected. However, they are also to the right of the Empa tests at an *a_p_-a_o_* value of 11.1 mm, which goes against the expected trend.

### 4.3. The Hartman-Schijve Master Relationship

The use of the Hartman-Schijve methodology (Equation (2)) is next examined to investigate whether the experimental results shown in Figure 2 and Figure 3 can be replotted to give a unique, linear, master curve so that the constants *D* and *n* may be ascertained. To achieve this, the values of *A* and $∆\sqrt{G_{thr}}$ are chosen so as to ensure that Equation (2) best fits an experimental set of test data of logarithmic *da*/*din* versus logarithmic $∆\sqrt{G}\;$over the entire range of FCG rates. In the tests undertaken by Empa and UCD, the values of *A* and $∆\sqrt{G_{thr}}\;$were determined using the ‘Total Least Squares’ methodology described in [41], so that, for a given set of test data at a given value of *a_p_*-*a_o_*, the logarithmic *da*/*dN* versus logarithmic $\left[\frac{∆\sqrt{G}\;-\;∆\sqrt{G_{thr}}}{\surd \left\{1\;-\;\sqrt{G_{max}}/\sqrt{A}\right\}}\right]\;$plots became virtually linear. After the individual linear relationship for a given set of test data points had been determined, a combined plot of each of the different tests was assembled, as shown in Figure 5. Here, it can be seen that, allowing for experimental error, all the resultant plots enable a single, linear, master relationship to be defined when using logarithmic axes. This unique, linear, master relationship may be readily fitted to all the twenty-five sets of data shown in Figure 5 and gives a value of the linear coefficient of determination, R^2^, of 0.975. Values of the slope, *n*, intercept, *D*, and mean value of $∆\sqrt{G_{thr}}\;$from fitting this master relationship are given in Figure 5 and in Table 1, and are subsequently used to compute the logarithmic *da*/*dN* versus logarithmic $∆\sqrt{G}$ relationships as a function of *a_p_-a_o_* and to predict the worst-case, upper-bound FCG rate curve, as described below. Thus, in other words, we have achieved one of the stated aims of the present paper, namely, to determine a similitude parameter ∆κ (see Equation (4)) such that, for a given value of ∆κ, the crack growth rate, *da*/*dN*, is independent of the pre-crack extension length *a_p_*-*a_o_*. As discussed in [11,42] and in the Appendix A, a similitude parameter is an essential requirement if laboratory-based tests are to be related to operational aircraft. Finally, it should be noted that clearly the nature of the adhesion at the interface between the carbon fibres and epoxy polymer matrix will play a role in the extent of fibre debonding that occurs [43,44] in the cyclic-fatigue tests.

### 4.4. The Computed FCG Curves 

Equation (2), together with the values of the parameters needed to fit the Hartman-Schijve relationship (see Table 1), can now be used to compute the full experimental curve of logarithmic *da*/*dN* versus logarithmic $∆\sqrt{G}$. The computed relationships are shown in Figure 2 and Figure 3 and, as may be seen, there is excellent agreement between the experimental data and the computed curves from the Hartman-Schijve methodology. Indeed, the values of the coefficients of determination, R^2^, associated with a comparison of the measured and computed curves shown in Figure 2 and Figure 3 were determined and have a mean of 0.89 and a standard deviation of 0.076. Therefore, despite the obvious and expected scatter in the experimental measurements, the values of R^2^ for the various computed curves are relatively high, thereby confirming the good agreement between the experimental and theoretical results.

### 4.5. The Worst-Case, Upper-Bound FCG Curve Deduced via the Hartman-Schijve Methodology

As explained above, the concept of an upper-bound curve for the FCG rate curve of the delamination is that such a curve is intended to give the worst case for the fatigue behaviour of the composite, since it excludes any retardation effects on the FCG rate, e.g., from fibre-bridging effects in the DCB test. Furthermore, it also should take into account the inherent experimental scatter typically observed from material and testing effects seen in such fatigue tests. This FCG rate curve should, therefore, act as an upper-bound curve to all the experimentally measured data for a composite and give a worst-case curve that can be used with confidence for industrial applications for (a) sound material development, characterisation, and comparison studies, and (b) accurate design and lifing studies. From the single, linear, master relationship shown in Figure 5, the parameters *D*, *n*, and $∆\sqrt{G_{thr}}\;$may be determined, as given in Table 1. The value of the parameter $∆\sqrt{G_{thr}}\;$to obtain the master relationship shown in Figure 5 is 10.53 ± 2.15 √(J/m^2^). The value of the constant, *A*, in Equation (2) is taken to be equivalent to the quasi-static value of the Mode I initiation fracture energy, $G_{co}$, at the onset of crack growth, which has the value of 250 ± 45 J/m^2^ [39,40]. As discussed above, the value of three times the standard deviation represents the allowable scatter for designing and lifing a single CFRP member where the loading is such that its failure would result in a loss of structural integrity of the component. Therefore, in the present paper, we have chosen to use the mean values of $∆\sqrt{G_{thr}}$ and $G_{co}$ minus three standard deviations to calculate the worst-case, upper-bound FCG curve. This FCG rate curve is shown in Figure 6 and indeed it may be seen to encompass, and bound, all the experimental data from both laboratories. Finally, it should also be noted that at an FCG rate of *da*/*dN* = 10^−2^ m/cycle, the slope of the curve is now very steep. In other words, at this point, the value of $∆\sqrt{G}$ is close to $\left(1-R\right)\surd G_{co}$, where the value of this term is 9.65 √(J/m^2^), taking the value of $G_{co}$ to be the ‘mean-3*σ*’ value (see Table 1), and the load ratio, R, of 0.1 as used for the present tests. As will be discussed below, this observation forms the basis for determining the scaling factor, SCF, that is used in Equation (5).

### 4.6. The Worst-Case, Upper-Bound FCG Rate Curve Deduced via the Simple-Scaling Methodology

**Figure 7:**
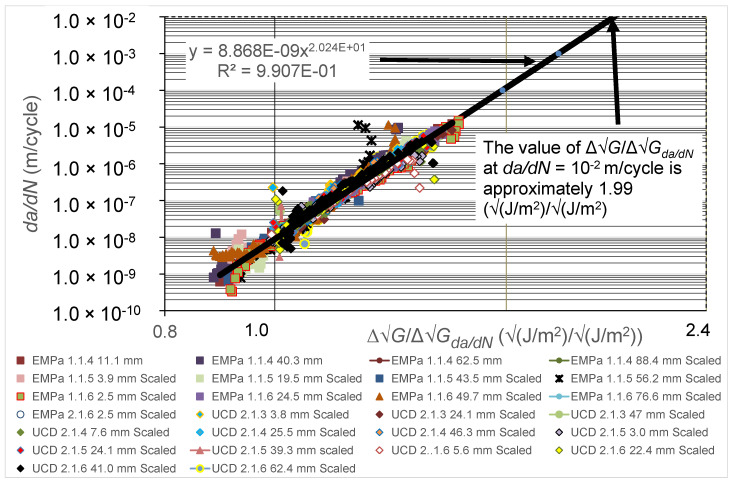
The logarithmic *da*/*dN* versus logarithmic $∆\sqrt{G}/∆\sqrt{G_{da/dN}}\;$normalised curves for the Empa and UCD test data. The values of $∆\sqrt{G_{da/dN}}$ used for each test are the values of $∆\sqrt{G}$ corresponding to a FCG rate of *da*/*dN* = 10^−8^ m/cycle as determined from Figure 2 and Figure 3 for the Empa and UCD experimental data, respectively. (Values are given in the legend for the pre-crack extension length, *a_p_-a_o_*, prior to the start of measurements from the DCB fatigue test).

As previously mentioned, the first step in determining the simple-scaling estimate for the worst-case FCG rate curve is to plot the logarithmic *da*/*dN* versus logarithmic $∆\sqrt{G}/∆\sqrt{G_{da/dN}}\;$curves (see Figure 7), where the values of $∆\sqrt{G_{da/dN}}$ used to normalise the *x*-axis in this figure are taken from Figure 2 and Figure 3. In Figure 7, we see that, when normalised in this fashion, the various curves essentially collapse to a single, unique curve that is, in a first approximation, independent of the value of *a_p_*-*a*_o_. Figure 7 also reveals that the resultant relationship between *da*/*dN* and $∆\sqrt{G}/∆\sqrt{G_{da/dN}}\;$can be approximated by using the following equation:

(6) $$da/dN=8.86\;\times \;10^{-9}.{\left(∆\sqrt{G}/∆\sqrt{G_{da/dN}}\right)}^{20.24}$$

In the second step, Equation (6) was then used to determine the value of the scaling factor, SCF, needed to ensure that at an FCG rate, *da*/*dN*, of 10^−2^ m/cycle, the value of $\Delta \sqrt{G^{*}}\;$was equal to the worst-case value of $\left(1-R\right)\surd G_{co}$, which had a value of 9.65 √(J/m^2^), as from Table 1. Using Equation (6), the value of $∆\sqrt{G}/∆\sqrt{G_{da/dN}}\;$at *da*/*dN* = 10^−2^ m/cycle was estimated to be 1.99 (see Equation (6) and Figure 7). Thus, from Equation (5), the value of the SCF is 4.85 √(J/m^2^), which is the value required for the SCF term to ensure that at *da*/*dN =* 10^−2^ m/cycle, the value of $\Delta \sqrt{G^{*}}\;$is 9.65 √(J/m^2^). (This value is given from Equation (5) as 9.65 √(J/m^2^) divided by 1.99). The rationale for this approach is that, as can be seen in Figure 6, at a crack growth rate of *da*/*dN* = 10^−2^ m/cycle, the slope of the FCG curve is very steep. Consequently, it follows that at *da*/*dN* = 10^−2^ m/cycle, the value of $∆\sqrt{G^{*}}$ should be close in value to the expression $\left(1-R\right)\surd G_{co}$ (= 9.65 √(J/m^2^)). 

The values of $∆\sqrt{G^{*}}$ associated with each of the UCD and Empa tests may now be computed from the normalised data shown in Figure 7 by taking the value of the scaling factor, SCF, in Equation (5) to be 4.85 √(J/m^2^). The resultant values of the logarithmic FCG rate, *da*/*dN*, are plotted versus logarithmic $∆\sqrt{G^{*}}$ in Figure 6. When inspecting Figure 6, it may be seen that the two different estimated worst-case curves as determined using the Hartman-Schijve and the simple-scaling methodologies are very similar, and that both do indeed bound the experimental data. The value of the worst-case fatigue threshold $∆\sqrt{G_{th}}$ at an FCG of 10^−10^ m/cycle obtained using the Hartman-Schijve methodology is approximately 4.70 √(J/m^2^) whilst that obtained using the simple-scaling methodology is approximately 3.89 √(J/m^2^) (see Figure 6). These two values are also quite similar.

To illustrate the effect of choosing a different crack growth rate at which to set the value of $∆\sqrt{G^{*}}$ to be close in value to $\left(1-R\right)\surd G_{co}$ (= 9.65 √(J/m^2^)), the approach described above was repeated but now for an FCG rate of 10^−3^ m/cycle. This yielded a value of the SCF of 5.43 √(J/m^2^). The corresponding estimated worst-case curve is also shown in Figure 6, where it is labelled as the ‘Curve AB’. This example aptly illustrates that both approaches to defining the FCG rate at which to take the value of $\left(1-R\right)\surd G_{co}$ yield fatigue thresholds and estimates of the worst-case FCG curve that are very similar to one another and are also very similar to those associated with the worst-case curve determined using the Hartman-Schijve methodology.

## 5. Conclusions

The present study has confirmed that in the fatigue crack growth (FCG) behaviour of a continuous fibre epoxy polymer matrix composite, as ascertained using the DCB test, a key experimental parameter is the value of the pre-crack (i.e., pre-delamination) extension length, *a_p_-a_o_*, in the DCB test specimen prior to any cyclic-fatigue measurements being undertaken. This phenomenon arises since varying the value of *a_p_-a_o_* typically leads to a varying degree of fibre bridging developing behind the tip of the fatigue delamination. The presence of such fibre bridging may retard the FCG rate and so lead to an impression of enhanced fatigue behaviour that is not present when no or very little fibre bridging occurs, as is typically the case in a real composite component. Also, the FCG results have been reproducibly measured by the two independent laboratories.

Furthermore, two data reduction methodologies are described for (a) computing the fatigue crack growth (FCG) rate, *da*/*dN*, curve, and (b) providing two different methods for determining estimates for the mandated worst-case FCG rate curve and the fatigue threshold limit below which no significant FCG occurs. These methodologies are based upon the Hartman-Schijve approach and a novel simple-scaling approach. These two different methodologies gave similar worst-case curves and both provided an upper bound for all the experimental data. The calculated FCG threshold values as determined from both methodologies were also in very good agreement. The simple-scaling approach has the advantage that it is independent of the Hartman-Schijve formulation and can be performed relatively easily. In future studies, it would be useful to examine if the methodologies proposed in the present paper can be extended to other values of the *R*-ratio and to other types of continuous fibre composites.

## Figures and Tables

**Table 1:** Parameters and values required to calculate, via Equation (2), the computed FCG rate curves (see Figure 2 and Figure 3) and the worst-case, upper-bound FCG rate curve shown in Figure 6. (Note: the units used to determine the values of *D* and *n* were m/cycle (*y*-axis) and √(J/m^2^) (*x*-axis); see Figure 5).

$∆\sqrt{G_{thr}}$ (√(J/m^2^))	Std. Dev., σ (√(J/m^2^))	$G_{co}$ (J/m^2^)	Std. Dev., *σ* (J/m^2^)	*D*	*n*
10.53	±2.15	250	±45	1.23 × 10^−10^	4.49

## Data Availability

The data will be made available at the completion of the project.

## Nomenclature

*a*	total crack (delamination) length, measured from the loading line
*a_o_*	length of the initial delamination in the DCB test specimen, i.e., the length of the (thin) film used as a starter crack, measured from the loading line
*a_p_*	length of the pre-crack (pre-delamination), measured from the loading line, in the test DCB test specimen prior to any cyclic-fatigue fracture measurements being taken
*a_p_-a_o_*	pre-crack (i.e., pre-delamination) extension length in the DCB test specimen prior to any cyclic-fatigue fracture measurements being undertaken
*A*	constant in the Hartman-Schijve equation
CFRP	carbon-fibre-reinforced plastic
*da*/*dN*	rate of fatigue crack (i.e., delamination) growth (FCG) per cycle
*D*	intercept in the Hartman-Schijve crack growth equation
DCB	double cantilever beam
Empa	Swiss Federal Laboratories for Materials Science and Technology
FCG	fatigue crack growth
*G*	energy release rate
$G_{co}$	quasi-static value of the Mode I (tensile) interlaminar fracture energy at the onset of crack growth
$G_{max}$	maximum value of the applied energy release rate in the fatigue cycle
$G_{min}$	minimum value of the applied energy release rate in the fatigue cycle
${G\prime }_{max}$	the value of $G_{max}$ at a crack growth rate of *da*/*dN* = 10^−10^ m/cycle
$G_{R}(a)$	quasi-static fracture energy as a function of the length of the propagating crack, a
∆G	range of the applied energy release rate in the fatigue cycle, as defined below $∆G=G_{max}-G_{min}$
$∆\sqrt{G}$	range of the applied energy release rate in the fatigue cycle, as defined below $∆\sqrt{G}=\sqrt{G_{max}}-\sqrt{G_{min}}$
$∆\sqrt{G_{th}}\;$	the value of $∆\sqrt{G}$ corresponding to an FCG rate of *da*/*dN* = 10^−10^ m/cycle
$∆\sqrt{G_{da/dN}}$	the value of $∆\sqrt{G}$ corresponding to an FCG rate of *da*/*dN* = 10^−8^ m/cycle
$∆\sqrt{G_{thr}}\;\;\;$	range of the fatigue threshold value of $∆\sqrt{G}$, as defined below $∆\sqrt{G_{thr}}=\sqrt{G_{thr.max}}-\sqrt{G_{thr.min}}$
$\sqrt{G_{thr.max}}$	threshold value of $\sqrt{G_{max}}$
$\sqrt{G_{thr.min}}$	threshold value of $\sqrt{G_{min}}$
$∆\sqrt{G^{*}}$	value of $∆\sqrt{G}$ for the worst-case FCG rate curve as calculated using the simple-scaling methodology
K	stress-intensity factor
$K_{max}$	maximum value of the applied stress-intensity factor in the fatigue cycle
$K_{min}$	minimum value of the applied stress-intensity factor in the fatigue cycle
ΔK	range of the applied stress-intensity factor in the fatigue cycle, as defined below $∆K=K_{max}-K_{min}$
LEFM	linear elastic fracture mechanics
*n*	exponent in the Hartman-Schijve crack growth equation
*N*	number of fatigue cycles
*NASA*	North American Space Administration
*P_max_*	maximum load applied during the fatigue test
*P_min_*	minimum load applied during the fatigue test
R	load ratio (= *P_min_*/*P_max_*)
*R^2^*	coefficient of determination
SCF	scaling factor
UCD	University College Dublin, Ireland
US	United States
USAF	United States Air Force
US DoD	United States Department of Defense
*δ*	displacement
Δ*κ*	crack driving force, i.e., the similitude parameter
*σ*	standard deviation

## Appendix A. The Relationship between $∆\sqrt{G_{th}}$ and $∆\sqrt{G_{thr}}$

The definition of the fatigue threshold term $∆\sqrt{G_{th}}$ can be traced back to the ASTM E647 [21] definition of the fatigue threshold ${\Delta K}_{th}$ for metals. In [21], the value of ΔK at an FCG rate *da*/*dN* = 10^−10^ m/cycle is arbitrarily defined as the fatigue threshold ${\Delta K}_{th}$; see [21] for more details. As such, $∆\sqrt{G_{th}}$ is defined as the value of $∆\sqrt{G}$ at an FCG rate *da*/*dN* of 10^−10^ m/cycle. However, it should be noted that, as delineated in Section 5.3 of MIL-STD-1530D [5], analysis is the key to the durability and damage tolerance assessment of an airframe, and the role of testing is to assist in validating and/or correcting the analysis. Furthermore, as explained in both the United States Joint Services Structural Guidelines JSSG-2006 [4] and the United States Air Force (USAF) MIL-STD-1530D [5], the airworthiness certification of a composite airframe must be based on a building-block approach that involves testing and analyses that range from the testing/analysis of laboratory-based coupons through to testing/analyses of representative sub-components, and finally, to full-scale aircraft. In this context, it should also be noted that USAF MIL-STD-1530D [5] states that the analyses should be based on linear elastic fracture mechanics (LEFM). Accordingly, in order to relate laboratory tests of representative components to a full-scale airframe, it is necessary that at each stage in the test, the crack-tip ‘similitude’ parameter in the laboratory test is equal to that in the airframe; see [11] for more details. This means that, for the airworthiness certification of a composite structure with delamination, it is essential to establish a valid crack similitude parameter. However, since as noted in CMH-17-3G [1], the design philosophy that is widely used is to adopt a ‘no growth’ design, the logical first step in moving away from this ‘no growth’ design philosophy is to allow for slow/limited delamination growth.

If we are to achieve this using the Hartman-Schijve equation given in the present paper, we need first to consider Equation (2) where we have the term$\;∆\sqrt{G_{thr}}$. Now, it is important to note that the term $∆\sqrt{G_{thr}}$ differs from the quantity $∆\sqrt{G_{th}}$, which corresponds to the value of $∆\sqrt{G}$ associated with a delamination growth rate, *da*/*dN*, of 10^−10^ m/cycle. However, the use of $∆\sqrt{G_{th}}$ in Equation (2) is inappropriate since, when $∆\sqrt{G}$ = $∆\sqrt{G_{th}}$, then Equation (2) will return a value of *da*/*dN* that is zero instead of the required value of *da*/*dN* = 10^−10^ m/cycle. Therefore, the term $∆\sqrt{G_{thr}}$ is introduced in Equation (2) to ensure that at $∆\sqrt{G}$ = $∆\sqrt{G_{th}},$ the value of *da*/*dN* is equal to 10^−10^ m/cycle. Hence, the values of $∆\sqrt{G_{thr}}$ and $∆\sqrt{G_{th}}$ are related by Equation (A1), viz:

(A1) $$D{\left[\frac{∆\sqrt{G_{th}}-∆\sqrt{G_{thr}}}{\sqrt{\left\{1-\sqrt{{G^{\prime }}_{max}}/\sqrt{A}\right\}}}\right]}^{n}=10^{-10}$$

where ${G^{\prime }}_{max}$ is the value of $G_{max}$ at *da*/*dN* = 10^−10^ m/cycle. Finally, the further relevance of the above discussion is that in the present paper it is shown that the term ∆κ, in Equation (4), acts as a valid similitude parameter.
